# Is conservation AI ready? Quantifying the challenges of automated species identification in Colombia

**DOI:** 10.3897/BDJ.14.e208137

**Published:** 2026-11-05

**Authors:** Gustavo Nicolas Paez Salamanca

**Affiliations:** 1 Data and Analytics Manager, UNICEF Asia Pacific Regional Office, Bangkok, Thailand Data and Analytics Manager, UNICEF Asia Pacific Regional Office Bangkok Thailand

**Keywords:** artificial intelligence, biodiversity monitoring, citizen science, iNaturalist, species identification, taxonomic bias

## Abstract

Multimodal artificial intelligence (AI) is increasingly adopted by conservation organizations to overcome taxonomic bottlenecks. However, its reliability may systematically fail for sparsely documented and recently described taxa. This study evaluates automated species identification using 10,000 research-grade iNaturalist images of Colombian vertebrates across amphibians, birds, fishes, mammals, and reptiles. Testing OpenAI GPT-5.2 with localized prompts but no taxonomic metadata, we found that species-level accuracy scales non-linearly with global photographic representation and decays severely for recently recognized taxa. At the 10th percentile of data availability, predicted accuracy collapsed to 6–12% for most groups, with mammals performing best at 41%. Furthermore, severe taxonomic hallucinations frequently crossed class boundaries, particularly among fishes and reptiles. These findings show that AI errors are not randomly distributed but are strongly associated with public photographic representation and taxonomic recency. While general-purpose AI offers immense value for rapid sorting, its operational integration requires strict safeguards, including forced abstention protocols, geographic filters, and mandatory expert escalation for rare or recently described species.

## Introduction

**Figure 1.**
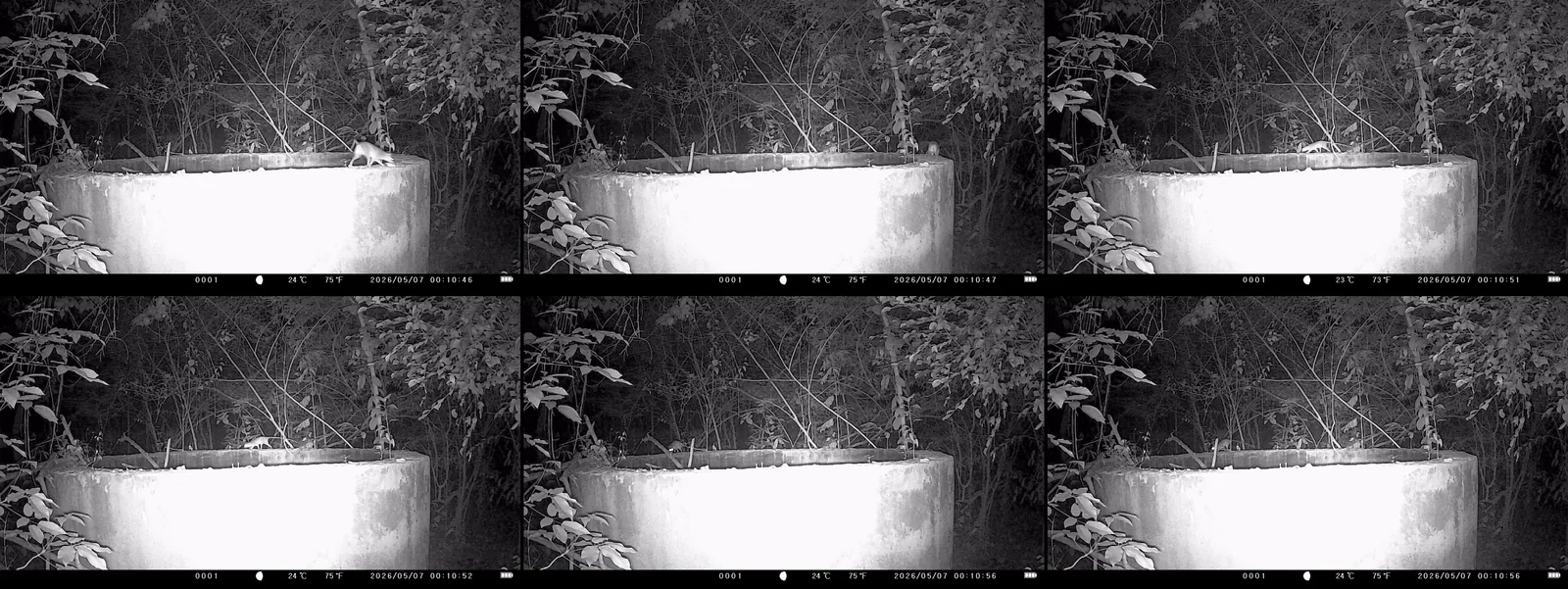
Camera-trap image recorded during biodiversity monitoring in the Morrocoy reserves in May 2026. A general-purpose multimodal AI system proposed *Bassariscus sumichrasti* despite receiving the Colombian locality and a description of the animal's locomotion, posture, facial structure, tail proportions, and possible colour pattern. Expert review identified the animal as *Metachirus myosuros*. We thank Dr Juan Fernando Díaz Nieto, Universidad EAFIT, for supporting the identification, and Fundación Sembrandopaz for the permission to use these images in the study.

The Asociación Sembrando Semillas de Paz (Sembrandopaz) conducts community-based conservation in Montes de María, Colombia, where armed conflict disrupted ecological research and left gaps in species inventories, distribution records, and ecological baselines (Murillo-Sandoval et al. 2021). In 2023, Fundación Crecer en Paz transferred more than 1,000 acres, including the Morrocoy 1 and 2 private protected areas near El Salado, El Carmen de Bolívar, to Sembrandopaz for conservation and restoration (Sembrandopaz 2023). These tropical dry forest remnants remain poorly surveyed. Camera traps deployed in May 2026 documented mammals and other terrestrial vertebrates, but reviewing thousands of images required scarce taxonomic expertise. ChatGPT was therefore used for preliminary identification. In one sequence, the identity of a small terrestrial mammal was uncertain. Despite a prompt specifying Colombia and morphological traits—including digitigrade posture, triangular face, binocular-looking eyes, a long horizontal tail, and possible banding—the AI suggested *Bassariscus sumichrasti*, whose documented range extends from southern Mexico through Central America to Panama, excluding Colombia (Pino et al. 2016). Fig. 1 compares the camera-trap record with AI and expert identifications. By favouring visual resemblance over geographic plausibility, the model risked introducing false records, misdirecting verification, and compromising biodiversity baselines.

Two further records illustrated distinct problems: *Coendou vossi*, described in 2025 following revision of the *C. quichua* species complex, occurs in the Colombian Caribbean but was assigned a superseded name still present in public resources (Ramírez-Chaves et al. 2025); *Cabassous centralis*, recognized for decades, has relatively few verified observations because of its fossorial and nocturnal habits (Chacón-Pacheco et al. 2019). Together with the geographically implausible *Bassariscus* identification, these cases illustrate three sources of error: ignored geographic constraints, outdated taxonomy, and sparse photographic representation. Each can produce plausible but incorrect biodiversity records.

Such errors have practical conservation consequences. Misidentifying a threatened, range-restricted, or recently described species as a common one can reduce the perceived conservation value of a site, affecting inventories, distribution maps, prioritization, and management decisions (Mace 2004, Rondinini et al. 2006). Even when an AI identifies the correct genus or nominal species, it may fail to recognize the biological unit that guides conservation action. For example, recent work argues that resident and Bigg's killer whales should be recognized as separate species because they differ in ecology, genetics, and conservation status (Morin et al. 2024). A generic identification of *Orcinus orca* therefore obscures information essential for management.

These examples raise a broader question: is general-purpose AI ready for conservation applications? Automated systems can substantially increase analytical capacity for organizations with limited resources, but their outputs influence ecological workflows that depend on accurate taxonomy, geographic context, and reliable baseline records. Species identification provides an ideal setting to evaluate these limitations because predictions can be compared directly against accepted taxonomic references.

This study evaluates species identification by a general-purpose multimodal AI using 10,000 images of Colombian vertebrates and standardized prompts. It tests three questions: whether accuracy increases with public photographic representation; whether recently described species are identified less accurately after accounting for that representation; and how far incorrect predictions depart from reference labels in the taxonomic hierarchy. Research-grade iNaturalist observations provide a measurable proxy for public visibility, not a reconstruction of model training data.

## Literature Review

### Automated identification in conservation monitoring

Camera traps extend wildlife monitoring to nocturnal, cryptic, wide-ranging, and infrequently detected vertebrates, but they also create a large image-review burden. Automated image analysis has reduced this burden through animal detection, species classification, individual recognition, and behavioural annotation (Burton et al. 2015, Tuia et al. 2022). Convolutional neural networks and camera-trap tools such as MLWIC2 and related classifiers can perform well when deployment conditions resemble the training data and the target species are represented in the label set (Norouzzadeh et al. 2018).

This performance is less stable under transfer. Background vegetation, infrared illumination, camera placement, occlusion, animal posture, and regional species composition can change independently of species identity (Beery et al. 2018). Specialist systems also depend on a predefined label space. An animal absent from that label set may be assigned to the most similar available category unless the workflow includes an unknown or abstention option. These limits matter most in inventories designed to discover which species occur at a site, and for rare or recently described taxa with few verified images. Specialist classifiers remain valuable, but their outputs are most interpretable when they are used on familiar taxa, comparable field conditions, and well-validated local data.

### General-purpose vision-language models

General-purpose vision-language models offer a different route to image identification. Contrastive image-text learning showed that visual and textual representations could be aligned across large datasets and then applied to categories outside a task-specific classifier (Radford et al. 2021). Current multimodal foundation models, including ChatGPT, Claude, and Gemini, extend this capacity by accepting images together with locality, habitat, behaviour, body size, colour pattern, or visible morphology (Bommasani and others 2021).

For small conservation organizations, the attraction is zero-shot access. An image can be submitted without building a local training set or specifying every possible taxon. The model can return a candidate identification and a textual justification, which may help triage backlogs and direct difficult cases to review. The same flexibility creates risk. A vision-language model predicts text from learned image-language associations; it is not a taxonomic authority, distribution database, or diagnostic key. It can confidently generate plausible but incorrect claims, a form of hallucination documented in generative systems (Ji et al. 2023). Geographic context in a prompt may influence the answer without strictly excluding impossible taxa, because these systems do not apply hard biodiversity constraints (Bubeck et al. 2023).

Commercial models also limit independent interpretation. Their training corpora, taxonomic balance, duplicate handling, geographic coverage, and treatment of outdated names are opaque (Bommasani and others 2021). Model updates can change predictions without revealing which data or parameters changed. Consequently, observed performance can be evaluated for a fixed set of images, prompts, and dates, but individual errors cannot be attributed confidently to a particular training record or internal representation.

### Unequal representation in biodiversity image data

Biodiversity image collections are highly uneven. Common, conspicuous, accessible, and frequently photographed species accumulate many records, while rare, nocturnal, fossorial, range-restricted, or recently recognized taxa remain in the long tail (Troudet et al. 2017). iNaturalist benchmarks and camera-trap studies show that species with fewer examples, less diverse imagery, or close visual relatives are harder to classify (Van Horn et al. 2018). High average accuracy can therefore hide low performance for the least represented taxa.

iNaturalist offers a reproducible way to describe this public information environment because it combines photographs, dates, locations, taxonomic labels, and community review in an open platform (Di Cecco et al. 2021). Yet its records are opportunistic rather than a designed inventory. Sampling is concentrated near roads, settlements, protected areas, tourism routes, and active observer communities, and taxa differ in detectability, public interest, and expert-verification capacity (Boakes et al. 2010). Research-grade status reduces some uncertainty but does not remove taxonomic, geographic, or observer bias (White et al. 2023). Record counts are therefore used here as a standardized proxy for public visual representation, not as abundance estimates or evidence of model-training exposure.

### Taxonomic change and digital representation

Taxonomic revision creates another form of information scarcity. A newly described species may have been photographed or collected for years under a broader species complex or a widespread relative. When the taxon is split, historical photographs, field guides, museum labels, and online observations do not immediately move to the new name, and some records cannot be reassigned because diagnostic evidence is absent. This creates a lag between formal recognition and digital representation under the accepted name (Patterson et al. 2010).

Taxonomic recency is distinct from low observation frequency. A recently described species may have many older records under another name, while a long-recognized species may remain poorly photographed because it is cryptic, nocturnal, uncommon, or geographically restricted. Both processes can lead an automated system toward a familiar umbrella label. For conservation, the severity of that error depends on the biological unit lost: a congener may be taxonomically close but still erase a restricted or threatened lineage.

### Accuracy, uncertainty, and ecological readiness

Overall accuracy, precision, and recall are useful but incomplete. They do not show whether errors concentrate among rare taxa, recent names, difficult images, or records with management consequences. Error severity is also uneven. Confusing species within a genus is not equivalent to assigning an image to another order or class, and even closely related taxa can differ in range, threat status, habitat, or legal protection (Deng et al. 2014).

Model-generated confidence does not solve this problem unless it is calibrated against observed accuracy. Modern neural systems can be poorly calibrated and overconfident when wrong (Guo et al. 2017). In applied conservation workflows, confidence language should support triage rather than replace verification. Systems designed for learning with rejection show why abstention is important: difficult or consequential records should remain unresolved until additional evidence is available (Cortes et al. 2016).

The broader question is whether ecological workflows are ready to use AI outputs as evidence. Mishra (Mishra 2026) argues that biased data, fragmented ecological records, uncertainty interpretation, and differences between ecological and AI expertise remain barriers. Species identification is a direct test of this problem because predicted labels can be compared with reference labels, public representation can be measured, and wrong predictions can be located in a taxonomic hierarchy. This study therefore evaluates not only whether the model is correct on average, but where its errors become most likely to require expert verification.

## Methodology

### Study design

This study evaluated general-purpose multimodal AI identification of research-grade iNaturalist animal photographs. Each image received predicted family, genus, and species labels, compared with its reference identification. Species-level correctness was examined against two predictors: global research-grade iNaturalist photographic record counts and formal-description year. Incorrect predictions were also assessed by taxonomic distance. Record counts represent observable public photographic representation, not confirmed model-training exposure; the analysis measures association, not whether particular images caused model outputs.

### Taxonomic and geographic scope

The sampling frame comprised vertebrates recorded in Colombia, divided into five operational groups: amphibians, birds, fishes, mammals, and reptiles. These categories support monitoring and comparison but are not five equivalent formal taxonomic classes. The fish frame included Characiformes, Siluriformes, and Cichliformes. Within each group, the study retained the three orders with the most eligible species in the Colombian iNaturalist frame.

### iNaturalist sampling frame and public-record measure

The iNaturalist API established eligibility through Colombian species-level observations with photographs and research-grade status, queried by target order using the platform’s Colombia place identifier. Species required at least one qualifying Colombian record. iNaturalist was selected because its extensive crowdsourced imagery provides a reproducible measure of public digital representation (Di Cecco et al. 2021).

Second, public photographic representation was measured globally. For every eligible species, the workflow counted all iNaturalist observations worldwide that met the same two filters: quality_grade = research and photos = true. The image submitted to the AI was also drawn from this global research-grade photo pool. Thus, Colombia determined which species were relevant, whereas the global count represented the amount of public photographic evidence available across the species' range. This design is appropriate for testing a general-purpose model because such systems may draw on associations learned from globally distributed image and text sources rather than from Colombian records alone (Radford et al. 2021).

Research-grade status was used because each sampled image required a reference identification that had passed iNaturalist's community data-quality process. It is not equivalent to a voucher-based expert determination and does not eliminate taxonomic error (White et al. 2023). However, evaluations of iNaturalist research-grade observations indicate that community consensus can provide reliable reference identifications at scale, especially when interpreted with awareness of taxonomic and observer bias (Mesaglio and Callaghan 2021). The global record count should therefore be read as a consistent index of digital presence, not as a complete census of online imagery or a guarantee of label correctness.

### Balanced multistage image sample

The final sample contained 10,000 photographs, with 2,000 images in each operational group. Sampling proceeded through the taxonomic hierarchy. Within the group currently furthest below its target, one of the three retained orders was selected with equal probability, followed by a genus selected with equal probability within that order. A species was then selected within the genus with probability proportional to its iNaturalist identification-agreement ratio, calculated as the summed number of identification agreements divided by the summed number of identification agreements plus identification disagreements across eligible global research-grade photographic observations. When agreement values were absent or summed to zero, species were selected uniformly. A random research-grade photograph was requested for the selected species and downloaded when a usable image URL was returned. Failed downloads were logged and replaced through continued sampling. The sampler used repeated random photograph requests from the selected species' eligible global research-grade photographic pool and did not implement an explicit without-replacement constraint.

**Figure 2.**
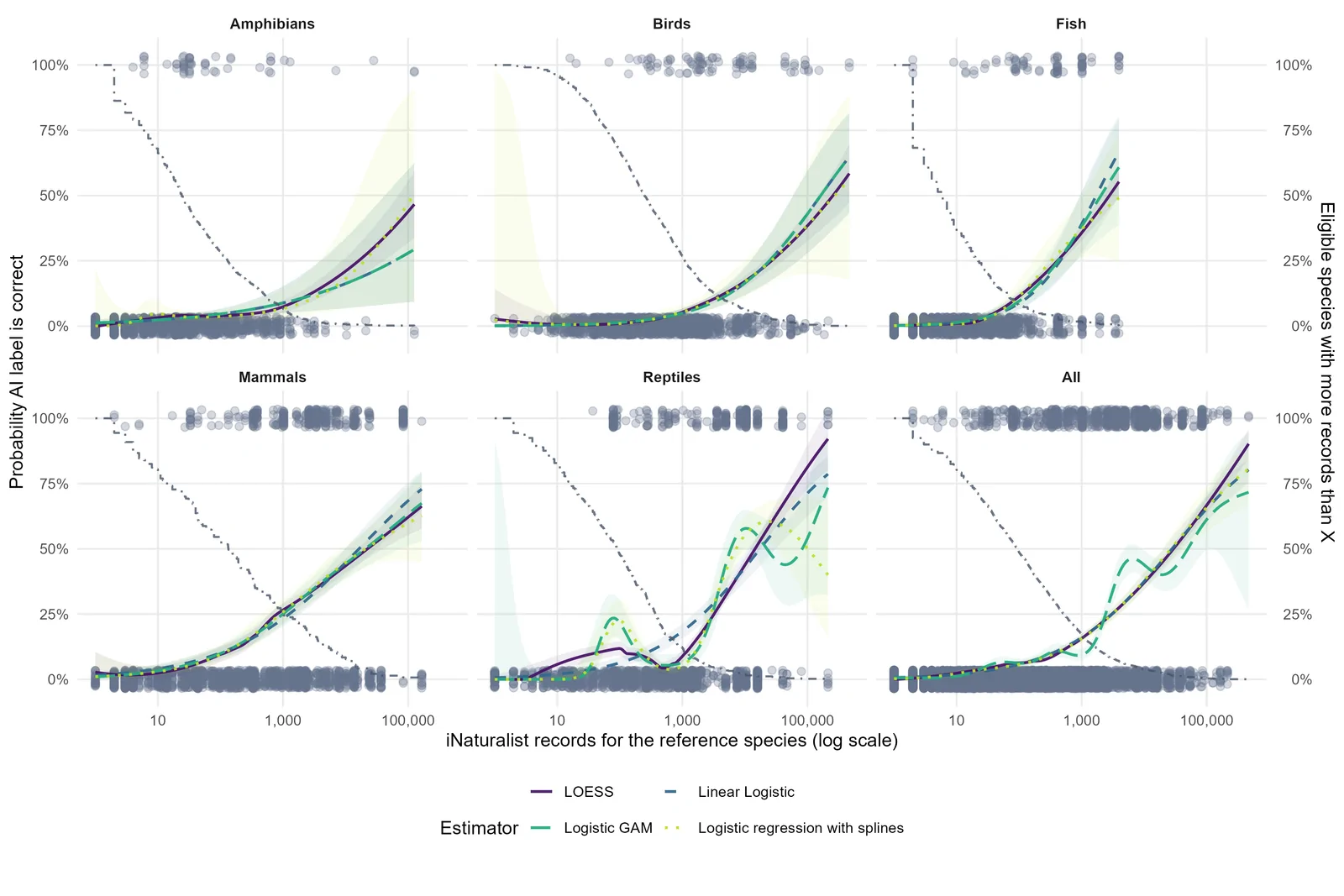
Confidence-weighted species-level identification accuracy in relation to global iNaturalist record availability. Points are image-level binary outcomes. Curves show LOESS, linear logistic regression, a logistic generalized additive model, and logistic regression with natural splines. The gray dash-dot curve is the percentage of the eligible species list exceeding each record-count threshold.

**Figure 3.**
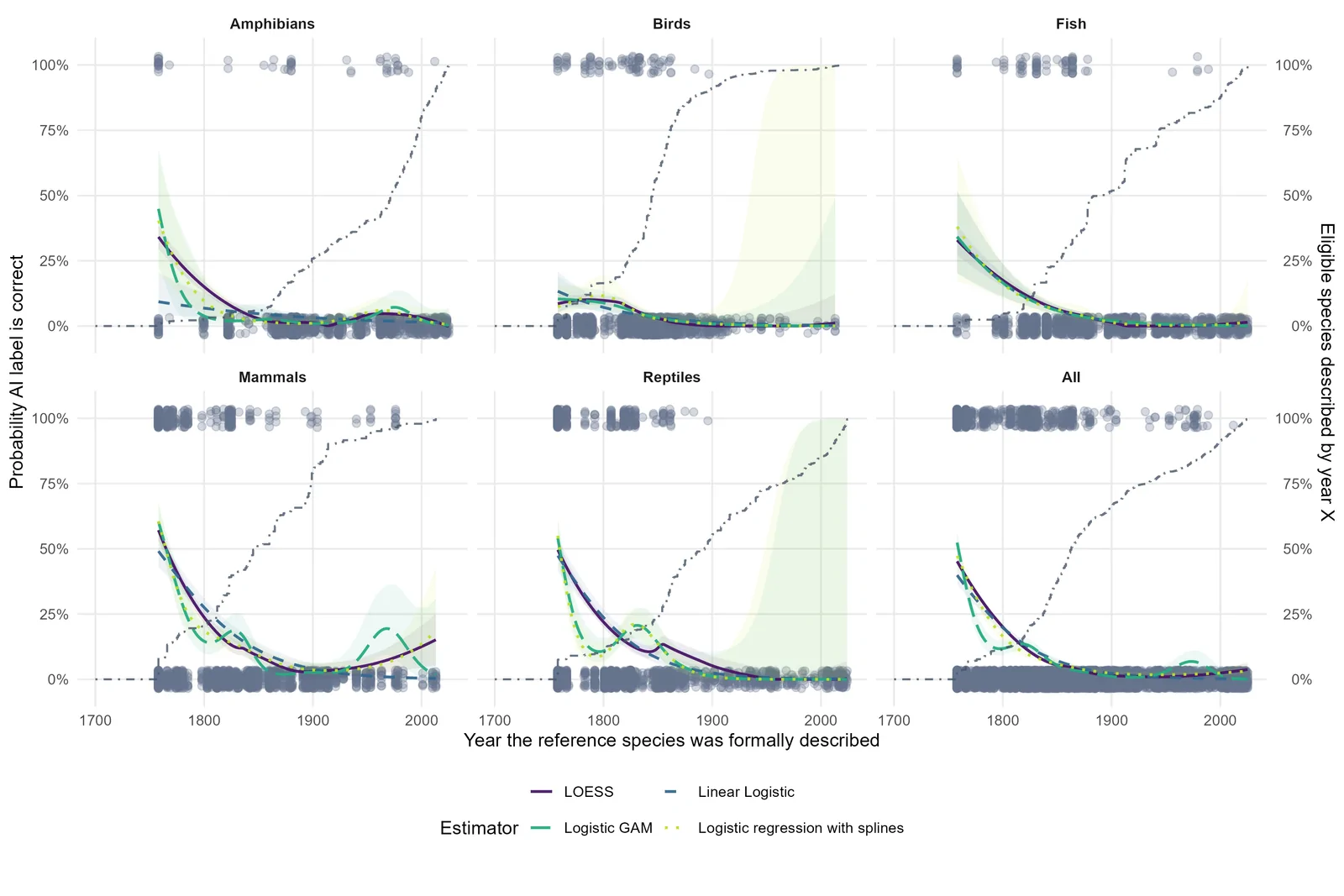
Confidence-weighted species-level identification accuracy in relation to formal-description year. Curves show the four estimators used in Figure 2. The grey dash-dot curve is the cumulative percentage of the eligible species list already described by each year.

This design deliberately prevents mammals, birds, or highly photographed species from dominating the evaluation. It therefore estimates performance under a balanced comparative design, not the expected accuracy of a random Colombian wildlife observation. As detailed in Table 1, sampling reached 893 of 955 eligible genera (93.5%) and 1,690 of 2,771 eligible species (61.0%). Species coverage was highest for mammals and fishes and lowest for birds and amphibians, reflecting differences in the number of eligible species per genus and the fixed 2,000-image allocation. For the distributional overlays in Fig. 2 and Fig. 3, the complete set of 2,771 eligible Colombian species identified during frame construction is termed the eligible species list. The right-hand axes report the percentage of this list above each record-count threshold or already described by each formal-description year.

### AI identification protocol

Images were submitted through the OpenAI Responses API using gpt-5.2. Original filenames were replaced with random alphanumeric identifiers, and images were re-encoded as JPEGs to remove embedded EXIF metadata, including coordinates, timestamps, and camera information. The output schema required family, genus, species epithet, confidence level, and a brief confidence explanation. Web-search preview was enabled, although retained metadata cannot establish its use in individual requests. Run-level records identify the model, prompt version, provider, run date, and parsed output; image-level provider-resolved snapshot identifiers were not retained. Reproduction remains subject to provider-side changes.

The model did not receive the iNaturalist taxon name, observation identifier, coordinates, date, order, genus, species, public-record count, formal-description year, or any metadata that directly revealed the reference answer. The prompt provided only the country context and the requested taxonomic resolution:

Identify the animal in this photo. The animal was seen in Colombia. Use visual evidence from the image and general biological knowledge. Return the most likely taxonomic family, genus, and species. If the exact identification is uncertain, still provide the most likely family, genus, and species, and set confidence to low or medium as appropriate. Do not leave fields blank.

Requiring complete family, genus, and species predictions prevented abstention, creating a forced-assignment stress test rather than an evaluation of selective identification. Operational systems should permit unresolved or coarser labels when species-level evidence is insufficient.

The country context made the task closer to an operational identification request and allowed the model to use broad biogeographic knowledge. At the same time, the sampled photograph could have been taken outside Colombia because images were drawn from the global pool. The prompt therefore supplied a valid species-occurrence constraint rather than the photograph's original locality.

The model assigned each answer a high, medium, or low confidence label. These labels were generated outputs, not sampling variables and not independently calibrated probabilities. They were retained because they indicate whether the model itself signalled uncertainty, but they were not treated as proof that a prediction was reliable.

### Reference labels and taxonomic matching

AI outputs were compared with the iNaturalist-derived reference label after standardizing capitalization, surrounding whitespace, and binomial structure. A genus prediction was correct when the predicted genus matched the reference genus. A species prediction was correct only when both the genus and species epithet matched the reference binomial. Family predictions were retained for error diagnosis.

Catalogue of Life provided the taxonomic backbone for accepted names, authorship, synonyms, and higher classification (Life 2025). This normalization followed the broader need to harmonize taxon names before comparing biodiversity data across sources (Grenié et al. 2023). It reduced false mismatches caused solely by formatting or synonymy. It could not resolve every disagreement among taxonomic authorities; ambiguous cases remain part of the study's label uncertainty and are addressed as a limitation.

### Formal-description year

Each sampled species was linked to Catalogue of Life/ChecklistBank using its accepted name. The authorship string and structured authorship fields were used to recover the year of formal description, with recent taxonomic acts checked against primary literature where necessary. Description year is an imperfect measure of taxonomic recency: an old name may have undergone a recent revision, and a newly delimited species may inherit historical records under a former concept. Nevertheless, it provides a reproducible indicator of the time available for a taxon to enter field guides, databases, image repositories, and public identification communities.

### Statistical analysis

The binary outcomes were genus-level and species-level correctness. The principal predictors were the global number of research-grade iNaturalist photo records for the reference species and formal-description year. Record count was analyzed on a logarithmic scale because it was strongly right-skewed and because a tenfold change in public representation is more interpretable than an equal arithmetic increment.

Four estimators were used to describe the relationship between each predictor and correctness. LOESS provided a local non-parametric curve. Linear logistic regression supplied a parsimonious monotonic benchmark. A logistic generalized additive model (GAM) allowed a smooth nonlinear response on the logit scale. Logistic regression with natural splines provided an intermediate level of flexibility. Agreement among these estimators was treated as evidence that a pattern was not an artefact of one functional form; divergence, especially near data-sparse tails, was treated as uncertainty rather than averaged away.

The displayed curves use analytic confidence weights of 1.00, 0.50, and 0.25 for high-, medium-, and low-confidence outputs, respectively. The scheme is an ordinal operational discount: high-confidence outputs retain full influence, medium-confidence outputs receive half that influence, and low-confidence outputs receive one quarter. These values were chosen to apply a transparent, progressively stronger penalty to self-reported uncertainty while retaining every image in the analysis. They are not calibrated probabilities, sampling weights, posterior probabilities, or estimates of the chance that an individual prediction is correct. An incorrect prediction remains incorrect under every weighting scheme.

A joint weighted logistic model assessed record availability, description year, and their interaction. The transformed predictors were centered and scaled before the interaction term was constructed so that coefficient magnitudes were numerically stable. The interaction tests whether the association between public records and accuracy changes across the taxonomic timeline. Because multiple images can represent the same species, the confidence weights are heuristic, and several class-specific tests are reported, coefficient tests are interpreted as exploratory evidence of association rather than causal effects or definitive hypothesis tests. No multiple-comparison adjustment was applied.

Two distributional reference points were used for readable summaries. For record availability, the reported threshold is the count exceeded by 90% of the eligible species list within a group; it therefore describes performance near the low-record portion of the species distribution. For description year, the reported threshold is the lower-tail year after which approximately 90% of the eligible species list was formally described. Equivalently, it is the 10th percentile of the description-year distribution, with small deviations possible because several species can share the same description year. These thresholds differ among groups and should not be interpreted as identical biological conditions.

### Cross-provider replication and reproducibility

Replication experiments were conducted with Anthropic Claude and Google Gemini using the same image set, country context, required output fields, and forced complete-assignment prompt. These experiments produced qualitatively similar principal relationships reported for OpenAI GPT-5.2: accuracy was consistently higher for better documented taxa and lower for recently described or sparsely represented taxa. The main article presents the OpenAI output set in full so that the figures, tables, and fitted estimators refer to one provider and one model identifier. OpenAI was selected as the primary reported provider because ChatGPT is widely used by non-technical practitioners and has become one of the most visible public interfaces for general-purpose generative AI (Chatterji et al. 2025). The repository contains provider-specific, checkpointed code that allows the same prompt, output schema, reference-label merge, and downstream analyses to be reproduced with Anthropic or Google models. The default identifiers in that code are claude-sonnet-4-6 for Anthropic and gemini-3.5-flash for Google, but runtime arguments can override them and write the exact identifier used to metadata. Full pseudocode for frame construction and image sampling is provided in the methodological appendix.

## Results

### Evaluation coverage and model-reported uncertainty

The balanced sample covered 893 of 955 eligible genera (93.5%) and 1,690 of 2,771 species (61.0%). Species coverage was highest for mammals (97.9%) and fishes (94.1%), followed by reptiles (60.3%), amphibians (53.7%), and birds (52.6%); the fixed 2,000-image allocation reduced proportional coverage in species-rich groups.

The API confidence field rarely labelled an answer as highly confident. Of 10,000 outputs, 154 (1.5%) were labelled high confidence, 8,357 (83.6%) medium confidence, and 1,489 (14.9%) low confidence (Table 2). Mammals accounted for 109 of the 154 high-confidence outputs (70.8%), despite representing only one-fifth of the images. This concentration anticipates the operational-group performance differences below: the model's uncertainty was not evenly distributed across vertebrate groups.

Across all 10,000 images, 948 identifications (9.5%) matched the reference species exactly at the binomial level. Unweighted exact species-level accuracy was 2.6% for amphibians (53/2,000), 4.5% for birds (90/2,000), 4.0% for fishes (81/2,000), 17.2% for mammals (345/2,000), and 18.9% for reptiles (379/2,000). These descriptive proportions are unweighted and are reported separately from the confidence-weighted fitted analyses below.

### Public photographic representation and species-level accuracy

Species-level identification improved as the number of global research-grade iNaturalist photo records increased, but the relationship was strongly nonlinear (Fig. 2). Across groups, fitted probabilities remained low throughout the sparsely documented portion of the record distribution and rose mainly once species had accumulated hundreds to thousands of public records. Mammals rose earlier and reached higher probabilities than the other groups. Amphibians, birds, fishes, and reptiles remained close to zero over a much larger part of the record range.

To contextualize performance for data-poor species, Table 3 evaluates predicted accuracy at the low-record threshold: the record count exceeded by 90% of the eligible species list within each group. Under LOESS, predicted species-level accuracy at this data-poor threshold was 5.8% for amphibians, 12.4% for birds, 9.6% for fishes, and 10.8% for reptiles. Mammals reached 41.0%, more than three times the bird estimate and more than seven times the amphibian estimate. The pooled LOESS estimate was 22.3%.

The comparison across estimators is as informative as the point estimates. Pooled predictions were nearly identical across methods (22.0–22.6%), and mammal predictions also clustered tightly (41.0–42.4%). Amphibian, bird, and fish estimates varied by only 1.2–2.3 percentage points. Reptiles were the exception: the linear logistic model predicted 19.8%, whereas the flexible estimators predicted 6.5–10.8%. The reptile relationship is therefore clearly positive at high record counts, but its value near the low- record threshold is sensitive to functional form and should not be summarized by the linear model alone.

These results show that record abundance is a central vulnerability rather than a minor refinement to performance. In the low-record region occupied by most eligible species, the model usually failed at exact species identification. The eventual rise at larger counts indicates that public representation and model performance are closely associated, but it does not establish that iNaturalist records themselves were used for training. In conservation workflows, this low-data failure mode is consequential: if a rare or range- restricted species lacks robust digital representation, an unverified AI output may replace it with a more familiar label and weaken the evidence base for a follow-up survey or protection.

### Formal-description year and species-level accuracy

Description year showed a second, complementary gradient (Fig. 3). Older species generally had higher fitted probabilities of correct identification, whereas predictions approached zero toward recently described taxa. The decline was particularly steep for mammals and reptiles. Fishes and amphibians also showed very low probabilities at the recent end of their distributions. Birds had a flatter curve over much of the twentieth century, but the model still performed poorly for the most recently described bird species.

Similarly to the record-count analysis, Table 4 evaluates performance at the lower-tail description-year threshold: the year after which the species comprising approximately 90% of the eligible list were formally described. At this threshold, LOESS estimates were 1.6% for amphibians, 10.1% for birds, 11.9% for fishes, 49.1% for mammals, and 23.9% for reptiles. The pooled estimate was 25.5%. The comparison is useful because it asks how the model performs at a point that still leaves most species in the more recent part of the taxonomic distribution. Mammals again stand out as the group with the highest fitted accuracy, while amphibians remain close to zero even at this relatively old cutoff.

The secondary axis in Fig. 3 should therefore be read cumulatively: at any year X, the gray curve gives the percentage of the eligible species list that had already been formally described by that year. The calendar thresholds are not directly comparable across groups. The year after which approximately 90% of eligible birds had been described was 1783, whereas the corresponding years were 1766 for mammals, 1796 for reptiles, 1821 for fishes, and 1864 for amphibians. The table therefore compares analogous lower-tail positions in each group's taxonomic distribution, not the same historical period. The figure still shows the later decline toward recently described taxa; the table summarizes the opposite reference point, where most of the eligible list lies to the right of the cutoff.

These temporal failures carry practical consequences. Recently described species often emerge from the splitting of historical species complexes. If an AI system fails to recognize a newly delimited, highly threatened endemic and instead predicts a widespread historical synonym or related taxon, it can mask the conservation significance of the record and provide a false sense of regional security.

### Joint association of records and description year

The joint weighted logistic models separated the average associations of log record availability and description year and tested whether those associations changed together (Table 5). A parametric logistic regression framework was used to estimate interpretable overall associations. The fitted model was:

\begin{varwidth}{50in}\begin{equation*}
            \operatorname{logit}\{\Pr(Y_i=1)\}=\beta_0+\beta_1 z[\log_{10}(R_i)]+\beta_2 z(D_i)+\beta_3 z[\log_{10}(R_i)]z(D_i).
        \end{equation*}\end{varwidth}

Here, \begin{varwidth}{50in}\begin{equation*}
            Y_i
        \end{equation*}\end{varwidth} denotes species-level correctness for image \begin{varwidth}{50in}\begin{equation*}
            i
        \end{equation*}\end{varwidth}, \begin{varwidth}{50in}\begin{equation*}
            R_i
        \end{equation*}\end{varwidth} is the number of global research-grade iNaturalist photographic records for the reference species, \begin{varwidth}{50in}\begin{equation*}
            D_i
        \end{equation*}\end{varwidth} is the formal-description year, and \begin{varwidth}{50in}\begin{equation*}
            z[\cdot]
        \end{equation*}\end{varwidth} denotes centering and scaling within the fitted group. Log record availability was positively associated with correctness for birds, fishes, mammals, reptiles, and the pooled sample; the amphibian coefficient was positive but not statistically distinguishable from zero. Description year was negatively associated with correctness for mammals, reptiles, and the pooled sample, but not for amphibians, birds, or fishes after adjustment for log record availability and the interaction.

For mammals, holding description year and the interaction structure constant, a one- standard-deviation increase in log public records was associated with approximately 198% higher odds of correct species identification [exp(1.094) ≈ 2.98]. Conversely, a one-standard-deviation shift toward more recently described mammal species was associated with roughly 70% lower odds [exp(−1.185) ≈ 0.31].

The interaction was significant for mammals (p = 0.001) and reptiles (p = 0.002), but not for amphibians or the pooled sample. The interactions for birds (p = 0.053) and fishes (p = 0.067) approached, but did not meet, the conventional 0.05 threshold. The positive mammal interaction indicates that the benefit associated with high log record availability was strongest in parts of the mammal taxonomic timeline where the model already performed better. The negative reptile interaction indicates a different structure: additional records did not offset taxonomic recency uniformly. Because these class-specific tests are exploratory and no multiple-comparison correction was applied, the robust conclusion is heterogeneity among groups rather than a universal interaction pattern.

### Taxonomic distance of species-level errors

**Figure 4.**
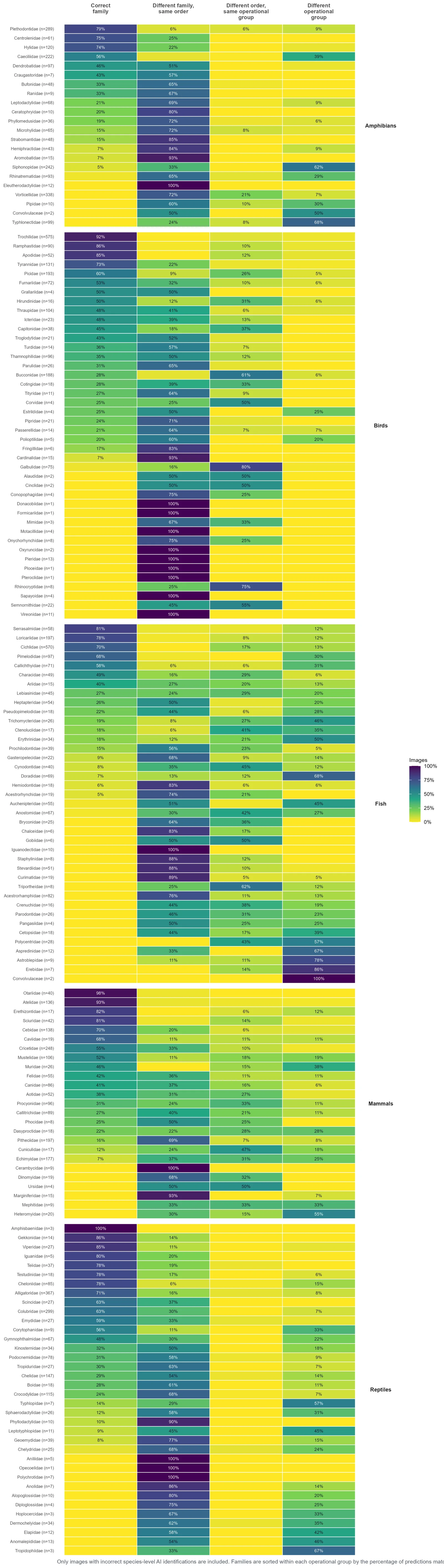
Taxonomic distance of predicted families among images with an incorrect species-level identification. The three panels show birds and fishes, amphibians and mammals, and reptiles. Cell values are percentages within each reference family and indicate whether the predicted family was the correct family, a different family in the correct order, a family in a different order but the same operational group, or a family in a different operational group. Blank or near-zero cells indicate little or no share in that category. Only images with an incorrect species-level prediction are included.

A wrong species label can still be taxonomically close, or it can cross family, order, taxonomic-class, or operational-group boundaries. Fig. 4 separates these outcomes by reference family. Among images with an incorrect species prediction, mammals retained the strongest family-level signal: the median share still assigned to the correct family was 31.2%. Birds and amphibians frequently remained within the correct order even when family was missed. Fishes had the weakest family retention, with a median same-family share of only 1.8%, and the highest median share assigned entirely outside the fish operational group (18.8%). Reptiles also showed substantial severe error, with a median share assigned to a different operational group of 12.7%.

**Figure 5.**
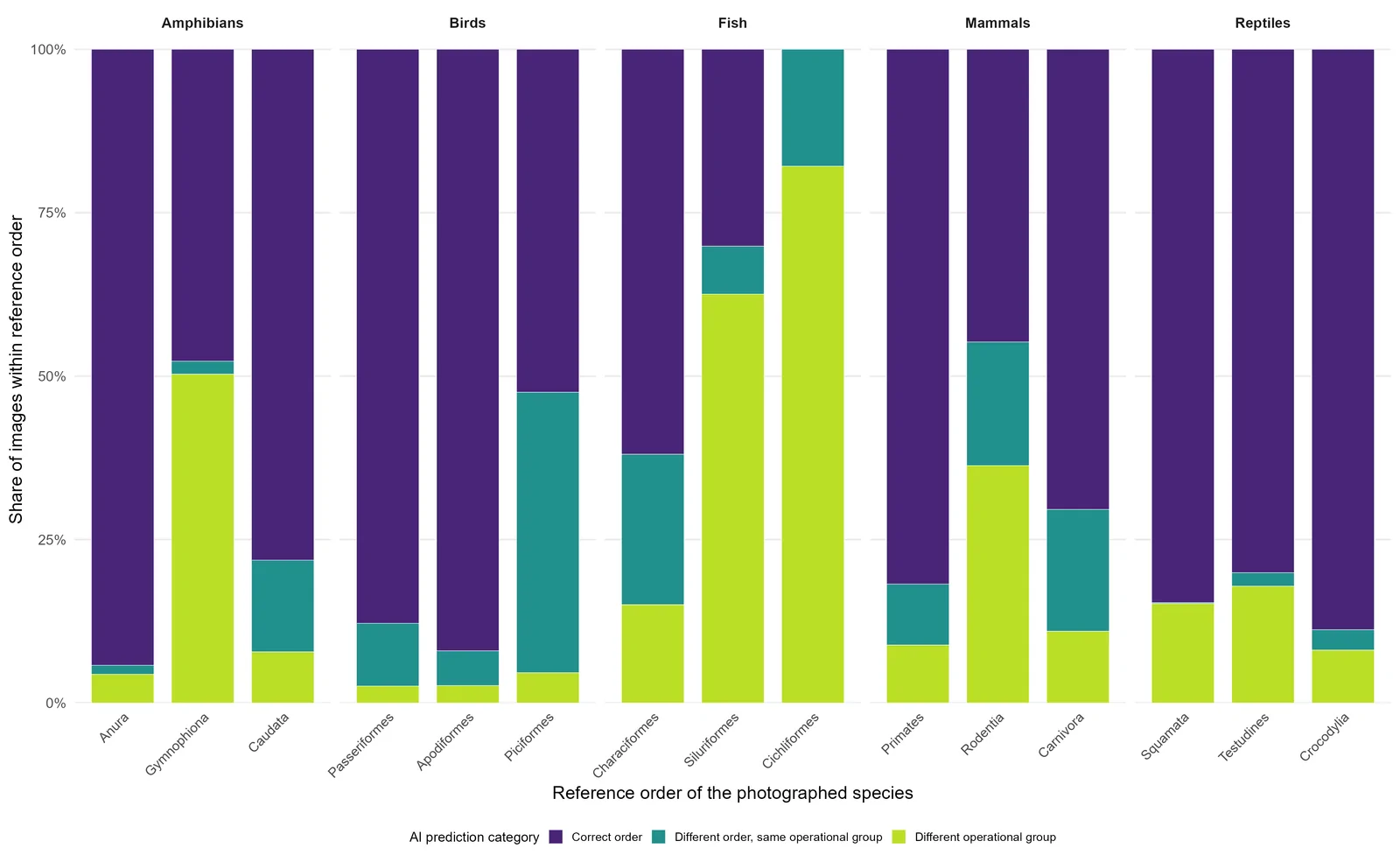
Taxonomic distance of inferred order predictions among images with an incorrect species-level identification. The model did not directly report order; predicted order was derived from the predicted family using the Catalogue of Life family reference. Each bar sums to 100% within the reference order.

The order-level summary (Fig. 5) confirms that error severity differs fundamentally by group. Mammal and bird errors were usually contained within the correct order or operational group. Amphibian errors were also often close at the order level, especially for Anura and Caudata. Fishes were the clearest counterexample: Siluriformes and Cichliformes had large shares of wrong-species images assigned outside the fish operational group. Reptiles were intermediate. Squamata and Crocodylia were often retained at order level, but Testudines still included a visible different-operational-group component.

The conservation implication is not captured by exact-species accuracy alone. A same- genus or same-family mistake may be recoverable during expert review, whereas predicting an entirely different operational group can redirect a survey, distort an inventory, or cause a reference record to be dismissed. These severe errors do not merely add noise; if accepted at face value, they can corrupt biodiversity inventories and misdirect targeted field surveys. The groups with the lowest species-level performance also include some of the most severe taxonomic errors, increasing the need for group- specific validation rules rather than one universal acceptance threshold.

## Discussion

### What the research shows

This research provides a practical test of general-purpose multimodal AI for conservation identification. Across 10,000 images, performance improved as species accumulated more public photographic records and generally declined toward recently described taxa. The central result is not simply that the model made mistakes, but that mistakes were structured: they concentrated where public reference material is sparse, taxonomy is changing, or visual discrimination is difficult. These are often the same cases in which conservation organizations most need specialist support.

The findings should not be interpreted as evidence that iNaturalist records were included in the model's training data. The study cannot observe a proprietary training corpus. Global research-grade iNaturalist records are used instead as a reproducible indicator of public visual representation. The positive record–accuracy association is consistent with the long-tail structure of biodiversity image data, where common taxa have many examples and rare taxa have few (Van Horn et al. 2018). Similar concerns appear across wildlife machine learning, where uneven training data, domain shift, and unrepresentative evaluation can translate into uneven operational performance (Tuia et al. 2022).

The results do not support rejecting AI identification altogether. A general-purpose model can provide provisional labels, sort backlogs, suggest likely families or genera, and help small organizations decide which images need urgent review. Its value is greatest when the decision is reversible, uncertainty is visible, and the output is treated as a hypothesis rather than a verified record.

### Why the most critical cases may be the hardest

Identification accuracy was lowest for taxa with few public records and for recently described taxa. This matters because uncommon, range-restricted, cryptic, juvenile, or recently delimited species may be both harder to identify and more consequential to miss. A false negative can suppress evidence of occupancy or range extension, while a confident false positive can create an apparent occurrence that redirects survey effort.

The taxonomic-distance results reinforce this point. Some wrong species predictions remained within the correct family or order and could plausibly be recovered during review. Others crossed class or operational-group boundaries, especially among fishes and reptiles. These are not only species-level inaccuracies; they can change how an observation enters an inventory and whether it reaches the appropriate expert.

Recently recognized taxa add another risk. When a former species concept is divided, images and descriptions may continue to circulate under the older name. An AI system may then return a familiar umbrella label even where the conservation question concerns a newly delimited lineage. Taxonomic revision is therefore not external to conservation; it changes the units used to estimate range, abundance, and threat (Mace 2004). As shown by the reassessment of eastern North Pacific killer whale ecotypes, even a broadly correct species-level name may be inadequate when management depends on populations, ecotypes, or evolutionary lineages (Morin et al. 2024).

### Implications for conservation workflows

A reasonable response to general-purpose model error is to prefer specialist systems. Specialist classifiers can be more accurate within a bounded domain, but they require verified training data, maintenance, and a relevant label set. Rare, recently described, and seldom photographed taxa have few such examples by definition. The practical answer is therefore a layered workflow rather than a choice between a general model and a perfect specialist model.

For small organizations, AI should first be used to generate candidate identifications. The model can propose a family, genus, species, and visible traits, but workflows should allow abstention or a coarser label when the image does not support species-level identification. Candidate names should then be screened against current country checklists, distribution sources, habitat, elevation, season, and recent synonyms. Catalogue of Life, iNaturalist, museum collections, regional checklists, and expert communities can support this process, provided their coverage and taxonomic limits are recognized (Mason et al. 2025).

Records should be escalated when they involve rare, threatened, newly described, out-of-range, trade-sensitive, or management-relevant taxa, or when geographic and diagnostic checks do not resolve the identification. An expert-review request should include the original image, date, appropriately generalized locality, habitat, scale or body-size information, candidate labels considered, and the reason the record matters. Every AI-assisted record should also retain an audit trail: image version, prompt, provider, model identifier, run date, structured output, reference sources, reviewer decision, and later taxonomic updates.

### Sensitive locations and data governance

AI identification also creates data-governance risks. Images may contain coordinates, timestamps, landmarks, people, property information, or other contextual clues. Uploading an unredacted image of a threatened or commercially valuable organism to a third-party service can disclose more information than is necessary for identification, while publishing precise coordinates can increase risks of disturbance or collection (Tulloch et al. 2018).

Before external submission, organizations should remove unnecessary metadata, mask human-identifying details, and share only the least precise locality sufficient for verification. Sensitive-species rules should distinguish among internal storage, expert sharing, public release, and delayed release. For highly sensitive records, local or institutionally controlled processing may be preferable.

### Limitations and future work

The analysis has several limits. First, iNaturalist is the sole empirical platform. Its research-grade photographs provide a large and reproducible frame, but they do not represent all internet imagery, museum material, camera-trap data, or field conditions. Research-grade status is a community data-quality designation rather than an independent voucher-based determination, and both labels and accepted names can change.

Second, global record counts were paired with a country-level prompt. This captures public imagery across each species' range but cannot distinguish global from Colombian representation. Opportunistic records are spatially uneven because sampling varies with accessibility, infrastructure, observer activity, taxon, and region (Boakes et al. 2010). The record–accuracy gradient should therefore be interpreted as an association with global public representation, not as a complete measure of regional visibility.

Third, forcing complete taxonomic responses prevents abstention, measuring forced-label performance rather than practical decision-support utility. Future work should evaluate both coverage and selective accuracy.

Fourth, the balanced design gives each operational group 2,000 images and restricts analysis to three species-rich orders per group. It supports comparison but does not estimate prevalence-weighted field accuracy and should not be generalized to untested orders or invertebrates. Description year is also a coarse proxy, meaning old names can be recently revised, and newly described species can be visually distinctive. Future work should incorporate revision dates, image quality, geographic coverage, photographer diversity, morphology, range size, and conservation status.

The principal figures present OpenAI GPT-5.2 to keep one complete output set in the main narrative. Replication analyses with Anthropic Claude and Google Gemini produced similar qualitative patterns in the associations of accuracy with photographic representation and description year. These checks do not establish numerical equivalence or architectural universality. Provider-specific code applies the same images, prompt, output schema, and downstream analysis so that interested researchers can reproduce the comparison.

Conclusions

General-purpose multimodal AI can speed wildlife-image sorting and generate candidate identifications. Although focused on Colombian vertebrates, the modular sampling, prompting, and analysis workflow can scale to other regions and taxa. Exact species accuracy was associated with public photographic representation and formal description year, and some incorrect labels moved beyond the reference order or operational group. The weakest performance occurred among sparsely documented or recently described taxa, where an incorrect label can obscure a restricted lineage or assign an observation to a locally implausible species.

The model's training data remain unknown. The present study therefore cannot determine the internal mechanism responsible for the observed associations. The study shows where identification becomes least reliable under a controlled design. AI can extend conservation capacity when it directs attention and expert time, but it should not replace taxonomic judgement for records with high conservation consequence.

### Statements and declarations

Acknowledgements: I thank Dr Juan Fernando Díaz Nieto (Universidad EAFIT) for his assistance with the identification of the *Metachirus myosuros* camera-trap record shown in Figure 1.

I also thank the Sembrandopaz conservation team, including Lillian Hall, Trevor Centeno, and the wider field and support team, for their dedication to biodiversity monitoring and conservation work in the Morrocoy protected areas. Their efforts made this research possible.

Disclaimer: The views and opinions expressed in this article are those of the author and do not necessarily reflect the views, policies, or positions of UNICEF or the United Nations.

Funding: No funding was received for this study.

Competing interests: The author declares no competing interests.

Author contributions: The author conceived the study, designed the methodology, developed the analytical workflow, collected and analysed the data, interpreted the results, wrote the manuscript, and approved the final version.

Data availability: The source biodiversity records are publicly available through iNaturalist and Catalogue of Life, subject to their respective terms and record-level licences. Reproducibility materials, including the sampling and analysis code, data dictionary, model-run documentation, and documented production parameters, are available on Zenodo (Paez Salamanca 2026). Source photographs are not redistributed. The exact 10,000-record sample manifest and linked analytical dataset are retained under controlled access because they preserve record-level linkages subject to licensing, sensitive-data, and redistribution conditions; access requests may be directed to the corresponding author as documented in the repository.

Code availability: The Python and R code used to collect, process, analyse, and visualize the data is publicly available in the GitHub repository at https://github.com/gnpaez12/ai-conservation-colombia. The version used in this study has been permanently archived as a software release in Zenodo (Paez Salamanca 2026). The archived repository includes reproducibility documentation, environment information, sampling parameters, and model-run metadata. Provider access and provider-side model changes may prevent exact regeneration of historical AI outputs.

Ethics approval: Ethics approval was not required because the study used publicly available biodiversity records and did not involve human participants, animals handled by the author, or private personal data.

Consent to participate: Not applicable.

Consent for publication: Not applicable.

Use of generative AI or AI-assisted technologies: Generative AI was used as an object of study to identify animal images and generate predictions evaluated against iNaturalist-derived reference labels. Outside the AI systems evaluated as the object of study, AI-assisted tools were used only for formatting and proofreading grammar and style.

## Methodological appendix

This appendix documents the frame-construction, sampling, and reproducibility procedures.

### Selection of target orders and construction of the species-record frame

The species-record frame was constructed as follows:

1. Define five operational groups: mammals, birds, reptiles, amphibians, and fishes.
2. For each group, query iNaturalist for species-level records from Colombia that contain photographs and have research-grade status.
3. Rank orders within each group by the number of eligible Colombian species and retain the three largest orders.
4. Resolve the retained order names to iNaturalist taxon identifiers.
5. For each retained order, query species counts using place_id = Colombia, quality_grade = research, and photos = true.
6. Use these Colombian records only to define the eligible species universe. Subsequent record counts and sampled photographs are global.
7. For every eligible species, query global iNaturalist observations using quality_grade = research and photos = true.
8. Aggregate the results by species, retaining operational group, order, genus, species, total research-grade photographic records, identification agreements, identification disagreements, and the derived agreement ratio.
9. Save a checkpoint after each API page so aggregation can resume after throttling, network failure, or interruption.

### Multistage image sampling

The balanced image sample was drawn with the following checkpointed procedure:

1. Set the total target to 10,000 images and the target for each operational group to 2,000 images.
2. Load the existing image-description checkpoint and count the images already sampled in each group.
3. Repeat the following steps until the total target is reached:
  1. Select the group with the largest deficit relative to its target; break ties at random.
  2. Select one retained order uniformly within that group.
  3. Select one genus uniformly within that order.
  4. Select one species within that genus with probability proportional to its iNaturalist identification-agreement ratio. If the ratios are missing or sum to zero, sample uniformly.
  5. Request a random photograph from the species' global research-grade photographic pool.
  6. If a usable URL is returned, download the image, assign a random local filename, and append its metadata to the checkpoint.
  7. If no photograph is returned or the download fails, log the attempt and continue sampling.
4. Write checkpoints regularly so the sampler resumes from the existing sample rather than restarting.

### Reproducibility materials and model-run metadata

Online Resources 1 and 2 provide the code and non-secret inputs required to reproduce the sampling frame, image-label merge, analyses, tables, and figures. The corresponding versioned software release is permanently archived in Zenodo (Paez Salamanca 2026). API keys are excluded. The principal components are:

1. inaturalist_top_colombia_orders.py, aggregate_inat_register_counts.py, and download_inaturalist_weighted_sample.py: construct the Colombian species frame, count global records, and draw the balanced sample.
2. identify_inaturalist_images_openai.py: submit images to the OpenAI Responses API with the reported prompt and save checkpointed predictions.
3. identify_inaturalist_images_robustness.py: apply the same protocol to Anthropic Claude and Google Gemini while recording provider, model, prompt version, request time, checkpoint status, and parsed outputs.
4. build_genus_species_reference_col.py and enrich_genus_species_reference_lineage_col.py: link sampled taxa to accepted names, authorship years, and higher taxonomy.
5. merge_ai_real_image_labels.py: combine reference labels, AI predictions, and record counts into the image-level analysis dataset.
6. The R scripts in Codes/R: regenerate the principal tables and figures.

Each model-run record retains, or should retain, the stable image identifier, complete prompt, provider, exact model identifier, endpoint or interface, UTC request time, relevant generation parameters, parsed family, genus, species, confidence, confidence reason, retry count, and error message. Provider-side model changes can prevent exact output reproduction; therefore, the model identifier and run date form part of the empirical record.

## Figures and Tables

**Table 1.** Sampling frame and final image sample. “Fishes” is an operational group; all sampled reference orders belong to Actinopterygii.

Group	Retained orders	List genera	Sampled genera	List species	Sampled species	Images
Amphibians	Anura, Gymnophiona, Caudata	85	85	547	294	2000
Birds	Passeriformes, Apodiformes, Piciformes	485	423	1260	663	2000
Fishes	Characiformes, Siluriformes, Cichliformes	162	162	290	273	2000
Mammals	Primates, Rodentia, Carnivora	74	74	143	140	2000
Reptiles	Squamata, Testudines, Crocodylia	149	149	531	320	2000
Total	15 orders	955	893	2771	1690	10000

**Table 2.** Model-reported confidence labels for the 10,000 identifications. Percentages are calculated within each operational group.

Group	High	Medium	Low	Images
Amphibians	6 (0.3%)	1450 (72.5%)	544 (27.2%)	2000
Birds	21 (1.1%)	1749 (87.5%)	230 (11.5%)	2000
Fishes	8 (0.4%)	1610 (80.5%)	382 (19.1%)	2000
Mammals	109 (5.5%)	1693 (84.7%)	198 (9.9%)	2000
Reptiles	10 (0.5%)	1855 (92.8%)	135 (6.8%)	2000
Total	154 (1.5%)	8357 (83.6%)	1489 (14.9%)	10000

**Table 3.** Predicted species-level accuracy at the low-data threshold, defined as the record count exceeded by 90% of the eligible species list. Values are probabilities; thresholds are group-specific.

Group	LOESS	Linear Logistic	Logistic GAM	Logistic Splines
Amphibians	0.058	0.077	0.077	0.056
Birds	0.124	0.112	0.112	0.121
Fishes	0.096	0.073	0.080	0.094
Mammals	0.410	0.415	0.418	0.424
Reptiles	0.108	0.198	0.074	0.065
All	0.223	0.222	0.226	0.220

**Table 4.** Predicted species-level accuracy at the lower-tail description-year threshold. For each group, this is the year after which approximately 90% of eligible species were formally described. Values are probabilities; thresholds are group-specific.

Group	Year	LOESS	Linear Logistic	Logistic GAM	Logistic Splines
Amphibians	1864	0.016	0.042	0.025	0.013
Birds	1783	0.101	0.093	0.097	0.109
Fishes	1821	0.119	0.109	0.109	0.104
Mammals	1766	0.491	0.447	0.486	0.478
Reptiles	1796	0.239	0.256	0.106	0.087
All	1789	0.255	0.243	0.128	0.221

**Table 5.** Weighted logistic models of species-level correctness using public photographic records, formal-description year, and their interaction. “Log records” denotes the centered and scaled base-10 logarithm of iNaturalist record counts; description year was also centered and scaled within each group before fitting. All values are rounded to three decimal places.

Group	Term	Estimate	SE	z	p
Amphibians	Log Records	0.296	0.210	1.409	0.159
Amphibians	Description Year	-0.220	0.223	-0.988	0.323
Amphibians	Log Records x Year	-0.145	0.154	-0.942	0.346
Birds	Log Records	1.393	0.230	6.061	<0.001
Birds	Description Year	-0.326	0.275	-1.185	0.236
Birds	Log Records x Year	0.377	0.195	1.937	0.053
Fishes	Log Records	1.203	0.231	5.216	<0.001
Fishes	Description Year	-0.149	0.312	-0.478	0.632
Fishes	Log Records x Year	-0.391	0.214	-1.830	0.067
Mammals	Log Records	1.094	0.153	7.132	<0.001
Mammals	Description Year	-1.185	0.185	-6.406	<0.001
Mammals	Log Records x Year	0.567	0.166	3.409	0.001
Reptiles	Log Records	0.572	0.151	3.791	<0.001
Reptiles	Description Year	-1.172	0.187	-6.285	<0.001
Reptiles	Log Records x Year	-0.433	0.137	-3.153	0.002
All	Log Records	0.866	0.095	9.096	<0.001
All	Description Year	-0.764	0.104	-7.372	<0.001
All	Log Records x Year	-0.100	0.077	-1.297	0.194
